# Genetic and non-genetic factors affecting morphometry of Sirohi goats

**DOI:** 10.14202/vetworld.2015.1356-1363

**Published:** 2015-11-28

**Authors:** S. D. Dudhe, S. B. S. Yadav, R. K. Nagda, Urmila Pannu, G. C. Gahlot

**Affiliations:** 1 Department of Animal Breeding & Genetics, College of Veterinary and Animal Science, Bikaner - 334 001, Rajasthan, India; 2 Principal Investigator, All India Co-ordinated Research Project on Sirohi Goats, Udaipur - 313 601, Rajasthan, India

**Keywords:** correlation, genetic, morphometric traits, non-genetic and Sirohi

## Abstract

**Aim:**

The aim was to estimate genetic and non-genetic factors affecting morphometric traits of Sirohi goats under field condition.

**Materials and Methods:**

The detailed information of all animals on body measurements at birth, 3, 6, 9, and 12 months of age was collected from farmer’s flock under field condition born during 2007-2013 to analyze the effect of genetic and non-genetic factors. The least squares maximum likelihood program was used to estimate genetic and non-genetic parameters affecting morphometric traits.

**Results and Discussion:**

Effect of sire, cluster, year of birth, and sex was found to be highly significant (p<0.01) on all three morphometric traits, parity was highly significant (p<0.01) for body height (BH) and body girth (BG) at birth. The h^2^ estimates for morphometric traits ranged among 0.528±0.163 to 0.709±0.144 for BH, 0.408±0.159 to 0.605±0.192 for body length (BL), and 0.503±0.197 to 0.695±0.161 for BG.

**Conclusion:**

The effect of sire was highly significant (p<0.01) and also h² estimate of all morphometric traits were medium to high; therefore, it could be concluded on the basis of present findings that animals with higher body measurements at initial phases of growth will perform better with respect to even body weight traits at later stages of growth.

## Introduction

Goat substantially contributes to the rural economy and provide livelihood to the poor sections. The total goat population of the country is 135.17 million,and it constitutes 26.46% of total livestock population.

Rajasthan ranks the first in total goat population of the country, i.e., 16.03% [1]. Among various genotypes available the Sirohi is one of the principal and renowned breed of goats. This breed has derived its name from Sirohi district of Rajasthan [2]. The source of income from this breed depends mainly on meat and milk production. In meat producing animals like sheep and goats, external body measurements could be a reliable indicator of its future performance with respect to live body weights, if and only if a correlation has been identified among these traits of interest [3]. Growth traits, which are available early, are a very important economic trait and could serve as an indicator for improvement of the traits that appear at the later age due to the association of body weights, and body measurements with fiber production [4].

In the present study, an attempt was made to study genetic and non-genetic factors affecting the body measurements along with genetic parameters.

## Materials and Methods

### Data

**Figure-1:**
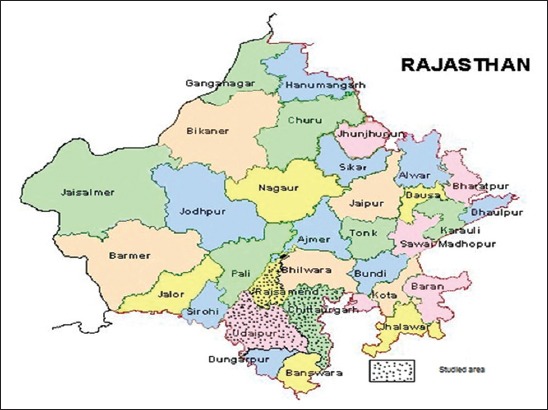
Study area map.

The detailed information of 3551 kids (male-1768 and female-1783) born during 2007-2013 on body measurements at birth, 3, 6, 9, and 12 months of age was collected from farmer’s flock under field condition, which were maintained under All India Coordinated Research Project (AICRP) on Sirohi goats, Livestock Research Station (LRS), Vallabhnagar, Udaipur, Rajasthan. Under this project, all Sirohi breeders were identified in the field. The study area (Figure-1) is located in western part of India and situated at 582 m above mean sea level on 24°35″ N latitude and 73°43″ E longitudes, which characterized with semi-arid climate with undulated topography having an average rainfall of 660 mm annually. Similarly, the temperature ranges from 2.3°C to 42.3°C. Breeding bucks properly tagged were reared and maintained at LRS under AICRP, Vallabhnagar during off breeding season and distributed to identified farmers during breeding season. Different bucks were rotated among farmers in different breeding seasons. The kids born out of such matings were tagged, and their pedigree records were maintained at LRS, Vallabhnagar. Animals were vaccinated against enterotoxaemia and peste des petits ruminants. The data were recorded on the same day for body weight and body measurements. The records were taken from birth up to 12 months of age at the interval of 3 months by the staff members of LRS. The data were classified into five clusters of three districts *viz*., (1) Vallabhnagar cluster of Udaipur district, (2) Railmagra, (3) Devgarh, (4) Nathdwara clusters of Rajasamand district, and (5) Bhadsoda cluster of Chittorgarh district, three seasons of birth *viz*. (1) rainy (July-October), (2) winter (November-February) and (3) summer (March-June), 6 years of birth from April month of the year up to March month of next calendar year, year 1 (2007-08), year 2 (2008-09), year 3 (2009-10), year 4 (2010-11), year 5 (2011-12), and year 6 (2012-13), five parity (1, 2, 3, 4, and ≥5), two types of birth:(1) single and (2) multiple and two sex:(1) Male and (2) female.

### Statistical methods

The data on growth trait was analyzed through mixed model least-squares and maximum Likelihood Computer Program PC 2, Harvey [5]. To study the effect of various genetic and non-genetic factors on body measurements the statistical model used was as under:


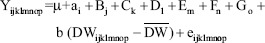


Where,

Y_ijklmnop_= Performance record of the p^th^ progeny of i^th^ sire belonging to j^th^ cluster, k^th^ season of birth, l^th^ year of birth, m^th^ parity, n^th^ type of birth and o^th^ sex.

µ = Overall population mean

a_i_ = Random effect of i^th^ sire

B_j_ = Fixed effect of j^th^ cluster (j= 1, 2, 3, 4, 5)

C_k_ = Fixed effect of k^th^ season of birth (k = 1, 2, 3)

D_l_ = Fixed effect of l^th^ year of birth (l = 1, 2, 3, 4, 5, 6)

E_m_ = Fixed effect of m^th^ parity (m= 1,2,3,4, ≥5)

F_n_ = Fixed effect of n^th^ type of birth (n = 1, 2)

G_o_ = Fixed effect of o^th^ sex (o = 1, 2)


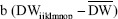
 = The regression of the trait on dam’s weight at kidding

e_ijklmnop_= Random error NID (0, σ^2^)

Duncan’s multiple range test as modified by Kramer [6] was used to make pairwise comparison among the least-squares means.

### Estimation of heritability

The heritability was estimated by sire component of variance-covariance obtained from the paternal half-sib analysis. The standard error of heritability was estimated as per Swiger *et al*. [7].

### Genetic and phenotypic correlations

The genetic and phenotypic correlations among traits were calculated from the analysis of variance and covariance among sire groups.

## Results and Discussion

The estimates of least-squares means of body height (BH), body length (BL) and body girth (BG) at birth, 3, 6, 9 and 12 months of ages are given in Tables-1-3, respectively.

**Table-1:** Least-squares means and SE for BH (cm) of Sirohi goat at different ages.

Factors	Traits				

	At birth	3 months	6 months	9 months	12 months
Overall mean (µ)	31.02±0.35 (3551)	51.53±1.19 (3073)	57.65±0.95 (2194)	61.76±2.24 (1642)	67.09±0.95 (1144)
Sire	**	**	**	**	**
Cluster	**	**	**	**	**
Vallabhnagar	29.16±0.38^a^ (278)	50.54±1.23^b^ (214)	55.52±1.04^b^ (123)	59.10±2.30^a^ (59)	59.54±1.65^a^ (36)
Railmagra	31.03±0.38^b^ (650)	47.35±1.22^a^ (593)	57.44±1.01^c^ (504)	60.71±2.27^b^ (396)	65.82±1.10^c^ (229)
Devgarh	31.70±0.38^c^ (1584)	52.63±1.22^cb^ (1439)	57.42±1.65^c^ (1184)	61.62±2.27^c^ (943)	64.76±1.12^b^ (715)
Nathdwara	29.38±0.61^a^ (37)	51.31±1.71^b^ (15)	53.42±1.65^a^ (12)	56.75±3.07^a^ (3)	-
Bhadsoda	33.85±0.38^d^ (1002)	55.81±1.22^d^ (812)	64.47±1.03^d^ (371)	70.63±2.28^d^ (241)	78.24±1.17^d^ (164)
Season	*	**	NS	*	**
Rainy	30.95±0.35^a^ (1303)	51.18±1.19^a^ (1171)	57.64±0.96 (795)	61.96±2.25^b^ (507)	67.61±0.96^b^ (377)
Winter	30.96±0.35^a^ (1723)	51.78±1.19^b^ (1443)	57.59±0.96 (1021)	61.96±2.25^b^ (829)	66.99±0.95^a^ (589)
Summer	31.16±0.36^b^ (525)	51.63±1.19^b^ (459)	57.71±0.97 (378)	61.36±2.27^a^ (306)	66.66±0.98^a^ (178)
Year of birth	**	**	**	**	**
2007-08	32.16±0.38^d^ (491)	53.21±1.22^d^ (453)	59.40±1.00^f^ (404)	63.92±2.27^d^ (350)	66.28±1.03^a^ (310)
2008-09	31.53±0.37^c^ (586)	50.98±1.22^b^ (530)	56.17±1.00^a^ (412)	61.36±2.27^b^ (316)	65.74±1.03^a^ (209)
2009-10	30.38±0.36^a^ (624)	50.54±1.20^a^ (525)	56.42±0.97^a^ (394)	59.82±2.25^a^ (320)	66.44±0.99^ba^ (186)
2010-2011	30.38±0.36^a^ (531)	51.25±1.20^b^ (467)	57.24±0.98^c^ (376)	61.24±2.26^b^ (308)	67.68±1.02^c^ (218)
2011-2012	30.91±0.36^b^ (668)	51.91±1.20^c^ (570)	58.90±0.98^e^ (406)	62.86±2.26^c^ (301)	69.31±1.02^d^ (221)
2012-2013	30.79±0.36^b^ (651)	51.29±1.21^b^ (528)	57.74±1.00^d^ (202)	61.37±2.32^b^ (47)	-
Parity	**	NS	NS	NS	NS
1^st^	30.97±0.36^a^ (278)	51.43±1.20 (664)	57.74±0.97 (507)	61.88±2.25 (393)	67.41±0.98 (276)
2^nd^	31.06±0.35^ba^ (650)	51.44±1.19 (589)	57.82±0.96 (455)	61.84±2.25 (348)	67.18±0.97 (243)
3^rd^	30.89±0.36^a^ (1584)	51.46±1.19 (553)	57.51±0.96 (411)	61.55±2.25 (303)	67.25±0.97 (220)
4^th^	30.99±0.36^a^ (37)	51.74±1.19 (461)	57.76±0.97 (317)	61.99±2.25 (218)	66.77±0.98 (152)
≥5^th^	31.20±0.35^ba^ (1002)	51.58±1.19 (806)	57.40±0.96 (504)	61.55±2.25 (380)	66.84±0.97 (253)
Type of birth	**	**	**	**	**
Single	32.12±0.35^b^ (2080)	52.58±1.19^b^ (1842)	58.70±0.96^b^ (1386)	62.46±2.24^b^ (1049)	67.74±0.95^b^ (752)
Multiple	29.93±0.35^a^ (1471)	50.48±1.19^a^ (1231)	56.60±0.96^a^ (808)	61.06±2.25^a^ (593)	66.44±0.96^a^ (392)
Sex	**	**	**	**	**
Male	31.41±0.35^b^ (1768)	52.49±1.19^b^ (1536)	58.51±0.96^b^ (1023)	62.68±2.24^b^ (679)	68.04±0.96^b^ (372)
Female	30.64±0.35^a^ (1783)	50.57±1.19^a^ (1537)	56.78±0.96^a^ (1171)	60.84±2.24^a^ (963)	66.14±0.95^a^ (772)
Regression on weight of dam at kidding	**	*	*	NS	**
Regression coefficient (b) (kg/kg)	0.066±0.015	0.068±0.031	0.076±0.037	0.046±0.048	0.157±0.057

Number of observations are given in parentheses. Estimates with different superscripts differ significantly.

** Highly significant (p<0.01),

* Significant (p<0.05), NS=Nonsignificant, SE=Standard error, BH=Body height

**Table-2:** Least-squares means and SE for BL (cm) of Sirohi goat at different ages.

Factors	Traits				

	At birth	3 months	6 months	9 months	12 months
Overall mean (µ)	28.29±0.39 (3551)	47.90±1.20 (3073)	53.96±1.01 (2194)	57.36±2.16 (1642)	62.65±098 (1144)
Sire	**	**	**	**	**
Cluster	**	**	**	**	**
Vallabhnagar	27.16±0.42^a^ (278)	48.79±1.25^b^ (214)	54.01±1.09^b^ (123)	56.85±2.22^b^ (59)	56.55±1.67^a^ (36)
Railmagra	28.65±0.42^b^ (650)	45.63±1.24^a^ (593)	55.48±1.08^c^ (504)	59.28±2.18^c^ (396)	63.90±1.13^c^ (229)
Devgarh	29.71±0.42^d^ (1584)	45.48±1.24^a^ (1439)	50.09±1.07^a^ (1184)	54.53±2.19^a^ (943)	58.07±1.14^b^ (715)
Nathdwara	27.00±0.66^a^ (37)	49.78±1.76^bc^ (15)	51.71±1.70^a^ (12)	51.36±3.05^a^ (3)	-
Bhadsoda	28.93±0.42^c^ (1002)	49.80±1.24^c^ (812)	58.51±1.09^d^ (371)	64.77±2.20^d^ (241)	72.07±1.19^d^ (164)
Season	**	**	NS	NS	NS
Rainy	28.21±0.39^a^ (1303)	47.57±1.20^a^ (1171)	53.98±1.01 (795)	57.60±2.16 (507)	62.74±0.99 (377)
Winter	28.13±0.39^a^ (1723)	48.06±1.20^b^ (1443)	54.12±1.02 (1021)	57.21±2.16 (829)	62.47±0.98 (589)
Summer	28.53±0.40^b^ (525)	48.06±1.21^b^ (459)	53.78±1.02 (378)	57.27±2.16 (306)	62.74±1.01 (178)
Year of birth	**	**	**	**	**
2007-08	29.62±0.42^e^ (491)	49.96±1.24^d^ (453)	55.68±1.06^e^ (404)	60.84±2.19^d^ (350)	64.47±1.06^e^ (310)
2008-09	28.98±0.41^d^ (586)	48.08±1.23^c^ (530)	52.82±1.05^b^ (412)	56.65±2.19^b^ (316)	60.90±1.06^a^ (209)
2009-10	27.80±0.40^b^ (624)	46.00±1.21^a^ (525)	52.13±1.03^a^ (394)	55.25±2.17^a^ (320)	61.65±1.02^b^ (186)
2010-2011	27.42±0.40^a^ (531)	47.63±1.22^b^ (467)	53.62±1.04^c^ (376)	56.50±2.17^b^ (308)	62.43±1.05^c^ (218)
2011-2012	27.82±0.40^b^ (668)	48.13±1.22^c^ (570)	55.31±1.03^e^ (406)	58.20±2.17^c^ (301)	63.78±1.05^d^ (221)
2012-2013	28.09±0.41^c^ (651)	47.58±1.22^b^ (528)	54.21±1.05^d^ (202)	56.72±2.24^b^ (47)	-
Parity	NS	NS	NS	NS	NS
1^st^	28.22±0.40 (278)	47.75±1.21 (664)	54.14±1.03 (507)	57.60±2.17 (393)	62.61±1.01 (276)
2^nd^	28.36±0.40 (650)	47.81±1.21 (589)	54.17±1.02 (455)	57.47±2.16 (348)	62.90±1.00 (243)
3^rd^	28.19±0.40 (1584)	47.90±1.21 (553)	53.79±1.02 (411)	57.26±2.16 (303)	62.79±1.00 (220)
4^th^	28.32±0.40 (37)	48.12±1.21 (461)	53.96±1.02 (317)	57.36±2.17 (218)	62.54±1.01 (152)
≥5^th^	28.37±0.40 (1002)	47.91±1.21 (806)	53.75±1.02 (504)	57.11±2.16 (380)	62.40±1.00 (253)
Type of birth	**	**	**	**	**
Single	29.37±0.39^b^ (2080)	48.83±1.20^b^ (1842)	55.06±1.01^b^ (1386)	58.07±2.16^b^ (1049)	63.20±0.98^b^ (752)
Multiple	27.21±0.39^a^ (1471)	46.96±1.20^a^ (1231)	52.86±1.02^a^ (808)	56.65±2.16^a^ (593)	62.09±0.99^a^ (392)
Sex	**	**	**	**	**
Male	28.62±0.39^b^ (1768)	48.71±1.20^b^ (1536)	54.72±1.01^b^ (1023)	58.32±2.16^b^ (679)	63.65±0.99^b^ (372)
Female	27.96±0.39^a^ (1783)	47.08±1.20^a^ (1537)	53.20±1.01^a^ (1171)	56.40±2.16^a^ (963)	61.65±0.98^a^ (772)
Regression on weight of dam at kidding	**	NS	*	NS	**
Regression coefficient (b) (kg/kg)	0.067±0.016	0.027±0.033	0.089±0.038	0.062±0.050	0.152±0.057

Number of observations are given in parentheses. Estimates with different superscripts differ significantly.

** Highly significant (p<0.01),

* Significant (p<0.05). NS=Nonsignificant, SE=Standard error, BL=Body length

**Table-3:** Least-squares means and SE for BG (cm) of Sirohi goat at different ages.

Factors	Traits				

	At birth	3 months	6 months	9 months	12 months
Overall mean (µ)	31.19±0.41 (3551)	51.62±1.18 (3073)	58.64±1.00 (2194)	62.26±2.35 (1642)	67.51±1.06 (1144)
Sire	**	**	**	**	**
Cluster	**	**	**	**	**
Vallabhnagar	29.69±0.44^b^ (278)	51.20±1.22^b^ (214)	56.08±1.09^a^ (123)	59.90±2.40^a^ (59)	59.66±1.73^a^ (36)
Railmagra	32.25±0.44^d^ (650)	48.82±1.21^a^ (593)	59.18±1.07^c^ (504)	62.45±2.37^c^ (396)	68.36±1.20^c^ (229)
Devgarh	32.02±0.44^c^ (1584)	52.90±1.21^c^ (1439)	57.72±1.06^b^ (1184)	61.84±2.37^b^ (943)	65.24±1.22^b^ (715)
Nathdwara	29.03±0.66^a^ (37)	50.34±1.70^ab^ (15)	56.81±1.77^ab^ (12)	57.43±3.15^a^ (3)	-
Bhadsoda	32.98±0.43^e^ (1002)	54.84±1.21^d^ (812)	63.43±1.08^d^ (371)	69.71±2.38^d^ (241)	76.77±1.26^d^ (164)
Season	**	**	NS	NS	**
Rainy	31.20±0.41^b^ (1303)	51.24±1.18^a^ (1171)	58.54±1.00 (795)	62.33±2.35 (507)	67.93±1.07^b^ (377)
Winter	31.02±0.41^a^ (1723)	51.85±1.18^b^ (1443)	58.63±1.00 (1021)	62.50±2.35 (829)	67.13±1.06^a^ (589)
Summer	31.36±0.42^b^ (525)	51.77±1.19^b^ (459)	58.76±1.01 (378)	61.96±2.35 (306)	67.46±1.08^a^ (178)
Year of birth	**	**	**	**	**
2007-08	32.82±0.43^d^ (491)	53.85±1.21^d^ (453)	60.62±1.05^e^ (404)	65.01±2.37^d^ (350)	67.34±1.14^b^ (310)
2008-09	31.95±0.43^c^ (586)	51.31±1.21^b^ (525)	57.80±1.05^a^ (412)	62.58±2.37^c^ (316)	66.02±1.13^a^ (209)
2009-10	30.37±0.42^a^ (624)	50.60±1.19^a^ (530)	57.37±1.01^a^ (394)	60.24±2.36^a^ (320)	66.90±1.10^b^ (186)
2010-2011	30.79±0.42^b^ (531)	51.19±1.19^b^ (467)	57.85±1.02^ba^ (376)	61.33±2.36^b^ (308)	67.89±1.12^cb^ (218)
2011-2012	30.72±0.42^b^ (668)	51.67±1.19^c^ (570)	59.68±1.02^d^ (406)	62.92±2.36^c^ (301)	69.40±1.13^d^ (221)
2012-2013	30.50±0.42^a^ (651)	51.09±1.20^b^ (528)	58.55±1.04^c^ (202)	61.51±2.42^b^ (47)	-
Parity	**	NS	NS	NS	NS
1^st^	31.06±0.42^a^ (278)	51.47±1.19 (664)	58.63±1.01 (507)	62.17±2.36 (393)	67.69±1.09 (276)
2^nd^	31.22±0.42^a^ (650)	51.44±1.18 (589)	58.73±1.01 (455)	62.31±2.35 (348)	67.64±1.08 (243)
3^rd^	31.11±0.42^a^ (1584)	51.58±1.18 (553)	58.57±1.01 (411)	62.09±2.35 (303)	67.48±1.08 (220)
4^th^	31.19±0.42^a^ (37)	51.84±1.19 (461)	58.88±1.01 (317)	62.65±2.36 (218)	67.32±1.09 (152)
≥5^th^	31.38±0.41^ba^ (1002)	51.76±1.18 (806)	58.41±1.01 (504)	62.11±2.35 (380)	67.40±1.08 (253)
Type of birth	**	**	**	**	**
Single	32.27±0.41^b^ (2080)	52.70±1.18^b^ (1842)	59.67±1.00^b^ (1386)	62.89±2.35^b^ (1049)	68.25±1.06^b^ (752)
Multiple	30.11±0.41^a^ (1471)	50.53±1.18^a^ (1231)	57.61±1.00^a^ (808)	61.64±2.35^a^ (593)	66.77±1.07^a^ (392)
Sex	**	**	**	**	**
Male	31.58±0.41^b^ (1768)	52.55±1.18^b^ (1536)	59.52±1.00^b^ (1023)	63.18±2.35^b^ (679)	68.46±1.07^b^ (372)
Female	30.80±0.41^a^ (1783)	50.69±1.18^a^ (1537)	57.77±1.00^a^ (1171)	61.35±2.35^a^ (963)	66.56±1.06^a^ (772)
Regression on weight of dam at kidding	*	*	NS	NS	**
Regression coefficient (b) (kg/kg)	0.039±0.015	0.071±0.031	0.037±0.041	0.008±0.048	0.153±0.058

Number of observations are given in parentheses. Estimates with different superscripts differ significantly.

** Highly significant (p<0.01),

* Significant (p<0.05). NS=Nonsignificant, SE=Standard error, BG=Body girth

### Effect of sire

The effect of sire was found to be highly significant (p<0.01) on all morphometric traits at birth, 3, 6, 9, and 12 months of age. The finding was in agreement with the observations of Kumar *et al*. [8] in Jamunapari goats and Karna *et al*. [9,10] in Cheghu goats. Tomar *et al*. [3] reported no significant effect of sire on the three morphometric traits.

Sire significantly affected the morphometric traits at all ages indicating the existence of additive genetic variability among these traits and significant influence of sire might be attributed to relative merits of the sires used.

### Effect of cluster

The effect of the cluster was found to be highly significant (p<0.01) on all morphometric traits at birth, 3, 6, 9, and 12 months of ages. Sharma *et al*. [11] observed highly significant (p<0.01) effect of cluster on BH and BL at birth and 3 months of age in Sirohi goats, BG at 3 months of age. However, Patil *et al*. [12] observed highly significant (p<0.01) effect on BG at 1 month of age, BH and BG at 6 months of age and BG at 9 months of age. Kharkar *et al*. [13] observed a significant effect on BL at birth. Gohain *et al*. [14] observed highly significant (p<0.01) effect of cluster on BG and BH in Assam local goats. Kuralkar *et al*. [15] reported highly significant (p<0.01) effect on all three measurements. Differences across clusters might be due to differences in grasses and herbage availability. Significantly (p<0.01) higher mean body measurements were observed in Bhadsoda cluster as compared with remaining four clusters in all age groups except BL at birth.

### Effect of year

The effect of year of birth was found to be highly significant (p<0.01) on all morphometric traits at birth, 3, 6, 9, and 12 months of ages. Present findings are similar with Tomar *et al*. [3] except at birth in BH in Sirohi goats, Sharma *et al*. [11] observed highly significant (p<0.01) effect of cluster on all three morphometric traits at birth and 3 months of age in Sirohi goats and Patil *et al*. [12] at 1 and 3 months of age on all three morphometric traits in Sangamneri goats. Karna *et al*. [10] reported the significant effect on all three traits at 3 and 6 months of age in Cheghu goats.

Higher values of all three morphometric traits were observed at birth in initial 2 years (2007-2008 and 2008-2009) then it remain almost constant. The BH declined upto 2^nd^ year and then again remained unchanged. The BG continuously declined up to 6 and 9 months of age. The highest BL was observed in the 1^st^ year of birth (2007-2008), whereas BH and BG in last year of birth (2012-2013). This might be due to the differences in climate, nutrition and management.

### Effect of season of birth

Influence of season of birth was highly significant (p<0.01) on BH at 3 and 12 months of ages, significant at birth and 9 months of ages and non-significant at 6 months of age. Highly significant (p<0.01) effect was observed on BG at birth, 3 and 12 months of ages, also on BL at birth and 3 months of ages. Similar results were also observed by Pathodiya *et al*. [16] in Sirohi goats. However, Sharma *et al*. [11] reported significant (p<0.01) effect of season of birth on BH and BL at birth, highly significant (p<0.01) effect on all three morphometric traits at 3 months of age. Barhat [17] reported highly significant (p<0.01) effect on all three morphometric traits in Marwari goats, Kharkar *et al*. [13] at 3 months on BL, 3 and 12 months BH and at birth and 12 months BG on Berari goats.

Summer born kids at birth have higher BH, BL and BG compared with rainy season and winter season. Kids born in rainy season attended maximum BH and BG.

### Effect of sex of kids

Sex of kids had highly significant effect (p<0.01) on all three morphometric traits at birth, 3, 6, 9 and 12 months of ages. However, the male kids were larger to females one with regards to their BH, BL, and BG at all the ages. These results are in agreement with the findings of Pathodiya *et al*. [16], Sharma *et al*. [11] reported highly significant (p<0.01) effect of sex of kid on BL and BG at birth in Sirohi goats and Reotheia *et al*. [18] reported highly significant (p<0.01) effect of sex of kid on BH and BG at 3 months of age and all three morphometric traitsat 6 and 9 months of age in Bakerwali goats. However, the non-significant effect of sex of kids on BH, BL and BG on all ages was observed by Kharkar *et al*. [13], whereas Kharkar *et al*. [19] significant effect on boy height at 12 months of age in Berari goats. Gohain *et al*. [14] observed highly significant (p<0.01) effect of sex of kid on all three morphometric traits in Assam local goats.

### Effect of type of birth

The type of birth had highly significant effect (p<0.01) on all three morphometric traits at birth, 3, 6, 9, and 12 months of ages. These findings were in agreement to those of Tomar *et al*. [3] at birth, 3 and 6 months of age in Sirohi goats and Patil *et al*. [12] at 1 and 3 months of age in Sangamneri goats on all three morphometric traits. Sharma *et al*. [11] reported highly significant effect (p<0.01) of type of birth on all three morphometric traits at birth, significant effect (p<0.01) on BH at 3 months of age. However, non-significant effect of type of birth on BH, BL and BG on all ages was observed by Kharkar *et al*. [13] in Berari goats.

Single born kids were larger in BH, BL and BG than those born as multiple at all the ages. This might be due to availability of more nutrients to the single born kid than those born in multiple births during pre and postnatal life.

### Effect of parity

The parity of dam had highly significant effect (p<0.01) on BH and BG at birth and at other ages non-significant effect of parity was observed. BL had non-significant effect of parity on all ages in the present study. Similar effect of dam’s parity on morphometric traits at all ages was reported by Nahardeka *et al*. [20] in Assam local goats. However, Pathodiya *et al*. [16] reported significant effect of parity of dam on all morphometric traits at birth in Sirohi goats.

### Effect of dam’s weight at kidding

The regression of dam’s weight at kidding had significant effect on BH at birth, 3, 6 and 12 months of ages, BL at birth, 6 and 12 months of ages and BG at birth, 3 and 12 months of ages. However, Kumar *et al*. [8] reported non-significant effect of dam’s weight at kidding on all morphometric traits at 6, 9, and 12 months of ages in Jamunapari goats and Sharma *et al*. [11] also reported non-significant effect on all morphometric traits at birth and 3 months of age.

### Genetic and phenotypic parameters for Morphometric traits at different ages

The results regarding estimated genetic and phenotypic parameters *viz*. heritability, genetic and phenotypic correlations of a population are presented in Tables-4-6.

**Table-4:** Estimates of heritability (on diagonal), genetic correlation (above diagonal) and phenotypic correlation (below diagonal) among BH at different ages in Sirohi goats.

Trait	BBH	3 BH	6 BH	9 BH	12 BH
BBH	0.693±0.130	0.621±0.059	0.533±0.102	0.636±0.080	0.767±0.074
3 BH	0.443±0.024	0.528±0.163	0.664±0.046	0.691±0.040	0.513±0.036
6 BH	0.354±0.026	0.658±0.017	0.709±0.144	0.520±0.030	0.664±0.049
9 BH	0.357±0.026	0.573±0.020	0.727±0.014	0.699±0.179	0.775±0.014
12 BH	0.333±0.026	0.495±0.022	0.557±0.020	0.741±0.013	0.708±0.188

BBH=Body height at birth, 3 BH=3 months body height, 6 BH=6 months body height, 9 BH=9 months body height, 12 BH=12 months body height

**Table-5:** Estimates of heritability (on diagonal), genetic correlation (above diagonal) and phenotypic correlation (below diagonal) among BL at different ages in Sirohi goats.

Trait	BBL	3 BL	6 BL	9 BL	12 BL
BBL	0.568±0.137	0.584±0.069	0.478±0.126	0.576±0.109	0.679±0.090
3 BL	0.373±0.025	0.408±0.159	0.660±0.050	0.550±0.052	0.648±0.030
6 BL	0.227±0.028	0.555±0.020	0.589±0.150	0.731±0.028	0.863±0.050
9 BL	0.261±0.028	0.513±0.022	0.703±0.015	0.571±0.172	0.835±0.026
12 BL	0.294±0.027	0.458±0.023	0.520±0.022	0.663±0.017	0.605±0.192

BBL=Body length at birth, 3 BL=3 months body length, 6 BL=6 months body length, 9 BL=9 months body length, 12 BL=12 months body length

**Table-6:** Estimates of heritability (on diagonal), genetic correlation (above diagonal) and phenotypic correlation (below diagonal) among BG at different ages in Sirohi goats.

Trait	BBG	3 BG	6 BG	9 BG	12 BG
BBG	0.590±0.147	0.519±0.058	0.620±0.088	0.508±0.061	0.608±0.061
3 BG	0.465±0.023	0.695±0.161	0.582±0.047	0.611±0.035	0.591±0.041
6 BG	0.340±0.026	0.559±0.020	0.563±0.138	0.667±0.023	0.900±0.044
9 BG	0.418±0.024	0.573±0.020	0.645±0.017	0.676±0.184	0.863±0.017
12 BG	0.394±0.025	0.506±0.022	0.497±0.022	0.668±0.017	0.503±0.197

BBG=Body girth at birth, 3 BG=3 months body girth, 6 BG=6 months body girth, 9 BG=9 months body girth, 12 BG=12 months body girth

#### Heritability

The heritability estimates for morphometric traits under study were of high magnitude. The heritability estimates for morphometric traits ranged among 0.528±0.163 to 0.709±0.144 for BH, 0.408±0.159 to 0.605±0.192 for BL, and 0.503±0.197 to 0.695±0.161 for BG. Higher estimates were also reported by Tomar *et al*. [3] at 3 and 6 months of age and Pathodiya *et al*. [16] at birth in Sirohi goats. On the other hand low heritability for BL and BH at 3 months and moderate heritability for morphometric traits at birth in Sirohi goats was reported by Tomar *et al*. [3]. The results indicated the presence of additive genetic variability and hence mass selection would be effective to improve these traits.

#### Genetic correlation

Estimates of genetic correlations between BHs at different ages ranged from 0.520±0.030 for 6-9BH to 0.775±0.014 for 9-12BH, between BLs at different ages ranged from 0.478±0.126 for BBL-6BL to 0.863±0.050 for 6-12BL and between BGs at different ages ranged from 0.508±0.061 for BBG-9BG to 0.900±0.044 for 6-12BG.

#### Phenotypic correlation

Phenotypic correlation is the association between phenotypic values of different traits measured on the same animal. It is a joint function of the genotype, and environment and interaction if any, between the two, but their relative contributions are varied. The estimates of phenotypic correlations between different morphometric traits at different ages are presented in Tables-4-6 and are discussed as follows.

Phenotypic correlations between BHs at different ages ranged from 0.333±0.026 for BBH-12BH to 0.741±0.013 for 9-12BH, between BLs at different ages ranged from 0.227±0.028 for BBL-6BL to 0.703±0.015 for 6-9BL and between BGs at different ages ranged from 0.340±0.026 for BBG-6BG to 0.668±0.017 for 9-12BG.

## Conclusion

The effect of sire was highly significant (p<0.01) and also h² estimate of all morphometric traits were medium to high; therefore, it could be concluded on the basis of present findings that animals with higher body measurements at initial phases of growth will perform better with respect to even body weight traits at later stages of growth. The estimates of phenotypic and genetic correlations were quite high in magnitude suggesting that improvement in BH would result in desired gain in body weight and body girth also.

## Authors’ Contributions

SBSY and RKN designed the experiment. SDD conducted the study and analyzed the data. UP and GCG revised the manuscript. All authors read and approved the final manuscript.
